# Dendrochronological Analysis of *Pinus pinea* in Central Chile and South Spain for Sustainable Forest Management

**DOI:** 10.3390/biology13080628

**Published:** 2024-08-17

**Authors:** Verónica Loewe-Muñoz, Antonio M. Cachinero-Vivar, Jesús Julio Camarero, Rodrigo Del Río, Claudia Delard, Rafael M. Navarro-Cerrillo

**Affiliations:** 1 Chilean Forest Institute (INFOR), Santiago 4811230, Chile; 2 Centro Nacional de Excelencia para la Industria de la Madera (CENAMAD), -ANID BASAL FB210015, Pontificia Universidad Católica de Chile, Santiago 7820436, Chile; 3 Laboratory of Dasometry and Forest Management, Forestry Engineering Department, School of Agriculture and Forestry, University of Córdoba, Edif. Leonardo da Vinci, Campus de Rabanales, 14071 Córdoba, Spain; o02cavia@uco.es; 4 Instituto Pirenaico de Ecología (IPE-CSIC), 50192 Zaragoza, Spain; 5 Research Group RNM-360, Departamento de Ingenieria Forestal, Evaluación y Restauración de Sistemas Agrícolas y Forestales (ERSAF), Universidad de Córdoba, 14014 Córdoba, Spain

**Keywords:** growth dynamics, natural forest, stone pine plantation, tree-ring analysis, stem diameter growth, aridity

## Abstract

**Simple Summary:**

Climate change will cause a reduction in the provision of goods and services of Mediterranean forests, including those of stone pine (*Pinus pinea*), an economically important species. We used a dendrochronological approach to address climate impact on the growth of stone pine natural stands and plantations. Our results indicate that increasingly arid conditions will affect both natural stands and plantations in native and exotic countries. Adaptive management will be essential to ensure the maintenance of the stands and their multifunctionality.

**Abstract:**

*Pinus pinea* is an important Mediterranean species due to its adaptability and tolerance to aridity and its high-quality pine nuts. Different forest types located in Mediterranean native and non-native environments provide the opportunity to perform comparative studies on the species’ response to climate change. The aims of this study were to elucidate growth patterns of the species growing in native and exotic habitats and to analyze its response to climatic fluctuations, particularly drought, in both geographical contexts. Understanding stone pine (*Pinus pinea*) growth responses to climate variability in native and exotic habitats by comparing natural stands and plantations may provide useful information to plan adequate management under climate change. By doing so, we enhance the understanding of *P. pinea*’s adaptability and provide practical approaches to its sustainable management. In this study, we reconstructed and compared the stem radial growth of seven stone pine stands, two in southern Spain and five in central–southern Chile, growing under different climatic conditions. We quantified the relationships between growth variability and climate variables (total rainfall, mean temperature, and SPEI drought index). Growth was positively correlated with autumn rainfall in plantations and with autumn–winter rainfall in natural stands. Growth was also enhanced by high autumn-to-spring rainfall in the driest Chilean plantation, whereas in the wettest and coolest plantation, such correlation was found in winter and summer. A negative impact of summer temperature was found only in one of the five Chilean plantations and in a Spanish site. The correlation between SPEI and tree-ring width indices showed different patterns between and within countries. Overall, exotic plantations showed lower sensitivity to climate variability than native stands. Therefore, stone pine plantations may be useful to assist in mitigating climate change.

## 1. Introduction

Forest plantations are crucial for maintaining the global terrestrial biomass, accounting for approximately half of it. These forests are subjected to climate change impacts, which are becoming increasingly complex and characterized by compound risks across regions [1,2]. Climatic stress negatively affects tree growth and survival [3,4,5] due to severe hydric deficits and increasing temperatures, as reported for economically important temperate conifers [6]. Unfortunately, forests in Mediterranean areas are particularly subjected to adverse weather events, including droughts [7]. That is the case of *Pinus pinea* L., commonly known as stone pine [8,9], an important Mediterranean species due to its adaptability and tolerance to aridity [10,11] and its high-quality pine nuts of commercial importance [12,13]. 

A better understanding of the impact of drought stress on *P. pinea* is necessary to inform adaptive forest management practices in a climate change scenario that will severely affect the provision of its goods and services [14]. The species has attracted substantial attention in dendrochronological studies in native countries [8,15], including Spain [16] where over 60% of the species’ forests are concentrated [17] and circa 500,000 hectares of plantations have been established [18]. However, despite the species’ increasing relevance in non-native countries, there is a notable disparity in research regarding its performance outside its native range. In particular, in central–southern Chile, the willingness for reforestation and the commercial potential of stone pine have led to the establishment of extensive plantations (5000 hectares since 2014); some of them are currently starting to produce cones. These plantations provide the opportunity to perform comparative studies on the dynamics of *P. pinea* growth and response to environmental conditions between Mediterranean native and non-native environments, characterized by unique ecological and climatic conditions, which directly influence the species’ growth dynamics [3,19]. 

In this study, a dendrochronology analysis was performed by comparing natural and planted forests in south Spain and planted forests in central–southern Chile. The aims of the study were to elucidate whether the growth patterns of the species differ between native and exotic habitats, discerning determinant environmental factors in both geographical contexts. By exploring these topics, we seek to not only enhance our understanding of *P. pinea*’s adaptability but also provide practical approaches to the sustainable management of stone pine. Thus, given the increasing global demand for pine nuts [20] and the urge for the ecological conservation of *P. pinea* stands, this study can be useful in informing forest management strategies, facilitating the optimization of plantation practices, and contributing to the broader discussion on sustainable forestry in areas with a Mediterranean climate. We hypothesize that growth patterns of *P. pinea* plantations in Chile differ significantly from those in Spain due to variations in environmental conditions and management practices, with Chilean plantations potentially exhibiting faster growth rates due to more favorable growing conditions.

## 2. Materials and Methods

### 2.1. Study Sites

**Figure 1:**
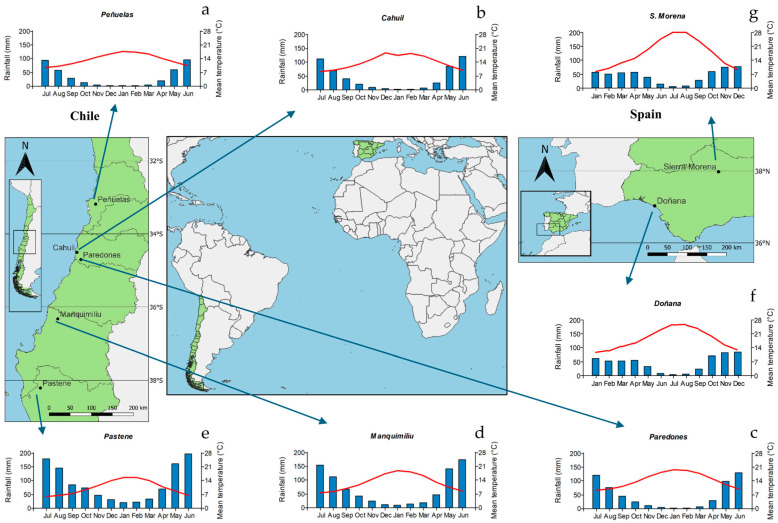
Site distribution showing the location of *Pinus pinea* stands and climate diagrams showing mean monthly temperature (red lines) and total monthly rainfall (blue bars) in Spain and Chile (both countries are shown in green). Abbreviations of sites (plantations): (**a**) CH1, Peñuelas (Chile); (**b**) CH2, Cahuil (Chile); (**c**) CH3, Paredones (Chile); (**d**) CH4, Manquimiliu (Chile); (**e**) CH5, Pastene (Chile); (**f**) SP1, Doñana (Spain); (**g**) SP2, Sierra Morena (Spain).

The study sites included in this research are located in south Spain and central–southern Chile. In Spain, one plantation (Parque Nacional de Doñana, SP1) and one natural stand (Sierra Morena, SP2) were selected. Stand spacing was 7 × 8 m (180 trees ha^−1^) and 6 × 6.5 m (280 trees ha^−1^), respectively. In Chile, five plantations (Peñuelas, CH1; Cahuil, CH2; Paredones CH3; Manquimiliu CH4; and Pastene, CH5) located from the Valparaíso to Araucania regions were selected; plantation spacing ranged from 3.5 × 3.5 m (833 trees ha^−1^) to 5 × 5 m (400 trees ha^−1^). Detailed information about stands, soil, and climate are presented in Table 1. The location of the plantations/stands and 10-year average climatic variables are presented in Figure 1. Climatic data were obtained from the Climate Research Unit dataset (CRU TS 4.03 [21]) and downloaded from the Climate Explorer webpage (http://climexp.knmi.nl/, accessed on 10 November 2024).

### 2.2. Dendrochronological Study

Two cores were collected at 1.3 m from 20 individuals in each site using Pressler increment borers. Cores were air-dried and sanded with sandpaper of increasing grain until ring boundaries were conspicuous. Then, they were visually cross-dated under the binocular scope and scanned at 1200 dpi (Epson Expression 10000XL). Tree-ring widths were measured with a 0.001 mm resolution along two radii per sample using the CooRecorder software version 9.8.1 [22]. Visual cross-dating was checked using the COFECHA software version 6.06P, which calculates moving correlations between individual tree-ring width series and the mean series of each site [23]. For Chilean plots, calendar dates were assigned to rings, following the Southern Hemisphere convention that assigns an annual ring to the calendar year in which the ring formation starts [24].

To calculate the individual tree diameter growth curves, the annual ring widths measured on two cores were averaged and summed for every tree from pith to bark. Then, a mean diameter curve was calculated for the seven sites and a sigmoidal function was fitted to characterize the relationship between tree age and *DBH* [25,26] as follows:

(1) $$DBH=\frac{a}{\left(1+{b\times e}^{-c\times age}\right)}$$

where *a*, *b*, and *c* are constants, and age is the tree’s age at 1.3 m estimated by counting the number of rings from bark to pith. 

Hereafter, tree-ring width data were transformed into basal area increment (BAI) which is biologically more meaningful to quantify growth variations between years and treatments, using the following formula:

BAI = π (R^2^_t_ − R^2^_t − 1_) (2)

where R corresponds to the tree radii and t is the year of tree-ring formation.

To analyze the BAI performance between forest types and countries, we used the Loess Regression, a non-parametric method commonly used to smoothen time series; in the graphical result, the shaded areas represent the higher and lower limits for the mean value of each forest type corresponding to each country [27]. Residual chronologies of the tree-ring width and tree-ring width index (RWI), were additionally computed (once the influence of long-term biological trends on radial growth was discarded due to increasing tree size and age by applying a detrending of the tree-ring series by fitting spline curves to each series, aided by the dplR [28] package in R software version 4.2.3 [29]). The resulting residual or pre-whitened individual series were averaged using a bi-weight robust mean to obtain the mean residual series of RWI for each site [30].

Several statistics were calculated for the best-replicated 1997–2022 period. This period was defined based on Expressed Population Signal (EPS) values and considering the period with EPS ≥ 0.85 as well replicated [5,31]. We calculated mean and standard tree-ring width values; the mean first-order autocorrelation of tree-ring widths (AC), which accounts for year-to-year persistence in growth; mean sensitivity (MS), which measures relative changes in growth indices between consecutive years; and mean correlation between series (Rbar), which is a measure of the coherence of the site chronology [32]. We also calculated mean basal area increment (BAI), mean basal area increment in the last 20 years (BAI_20_), and cumulative radial growth (CG). Lastly, an analysis of variance (ANOVA) was used to compare log (x + 1)- transformed data of BAI, BAI_20_, and CG. Three factors were considered in these comparisons: country, forest type (natural vs. planted stand), and site.

### 2.3. Climate Data and Drought Indices

**Figure 2:**
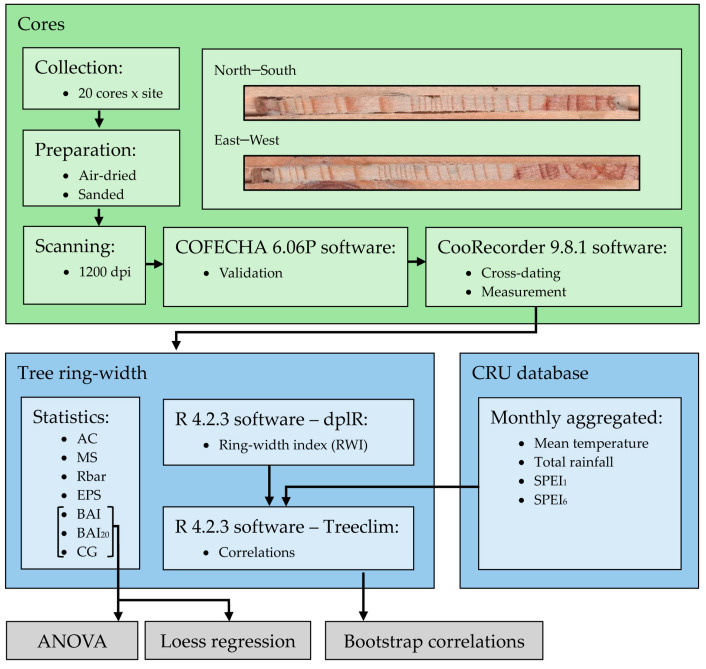
Methodological workflow representation.

We used monthly 0.5°-gridded data from the Climate Research Unit dataset (CRU TS 4.03 [21]), which were collected from the Climate Research Unit Time-Series version 4.07 (CRU TS) climate dataset (https://crudata.uea.ac.uk/cru/data/hrg/cru_ts_4.07/cruts.2304141047.v4.07/, accessed on 10 November 2023). The monthly CRU climate data (mean temperature and total rainfall) were complemented and compared with rainfall data from the “Climate Hazards Group Infrared Precipitation with Stations” dataset [33]. We acknowledge that using local climate series may render more specific results regarding climate–growth relationships; however, the available local series were not comparable given their different lengths and data gaps. We used the 0.5°-gridded Standardized Precipitation Evapotranspiration Index (SPEI) downloaded from https://monitordesequia.csic.es/, (accessed on 10 November 2023) at scales of 1 (SPEI_1_) and 6 (SPEI_6_) months to characterize drought severity [34]. The SPEI is computed as a cumulative climatic water balance calculated at several temporal scales. Mean RWI series or chronologies were related to monthly climate data (mean temperature and total rainfall) and drought indices (SPEI_1_ and SPEI_6_) using the Treeclim R package [35] and reported as bootstrap correlation coefficients [36]. A summary of the methodological workflow is presented in Figure 2.

## 3. Results

### 3.1. Dendrochronological Statistics and Growth

**Figure 3:**
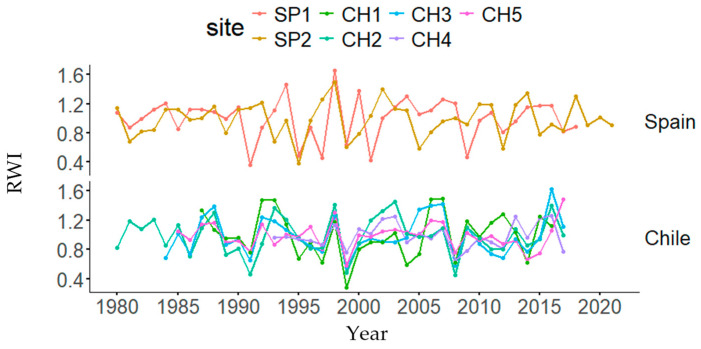
Tree-ring width index (RWI) changes by country (Spain and Chile) and site (Doñana, SP1; Sierra Morena, SP2; Peñuelas, CH1; Cahuil, CH2; Paredones, CH3; Manquimiliu, CH4; Pastene, CH5).

Graphical representations of each tree-ring width index and DBH decreases or gains in both natural and planted stands are presented in Figure 3 and Figure S1, respectively. Dendrochronological statistics are presented in Table 2. The highest and lowest mean BAI and BAI_20_ values were found in the SP1 and CH1 (Chile) plantations, respectively. The highest AC was found in CH2, the highest MS was found in SP1, and the highest EPS was found in CH5. In the ANOVAs, country and forest type were statistically significant for BAI, whereas site was also significant for BAI_20_ and only country was significant for CG (Table S1).

### 3.2. Growth Responses to Climate

**Figure 4:**
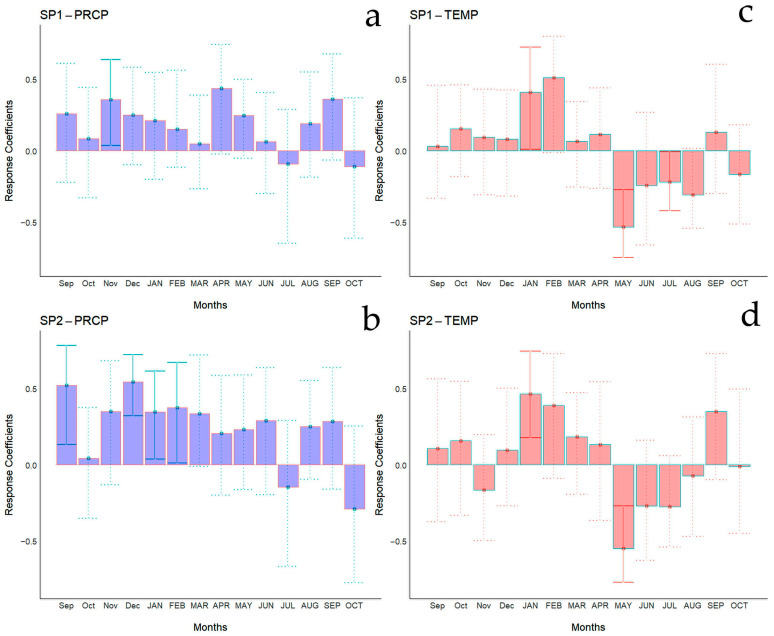
Bootstrapped correlations relating tree-ring width indices to monthly climate variables (PRCP, total rainfall—(**a**,**b**); Temp, mean temperature—(**c**,**d**)). Correlations were calculated from the previous September to the current October, and significant (*p* < 0.05) values are shown with continuous error lines. Spanish stands: SP1 (Doñana) and SP2 (Sierra Morena). Monthly climatic variables from the previous and current years are abbreviated with lowercase and uppercase letters, respectively.

**Figure 5:**
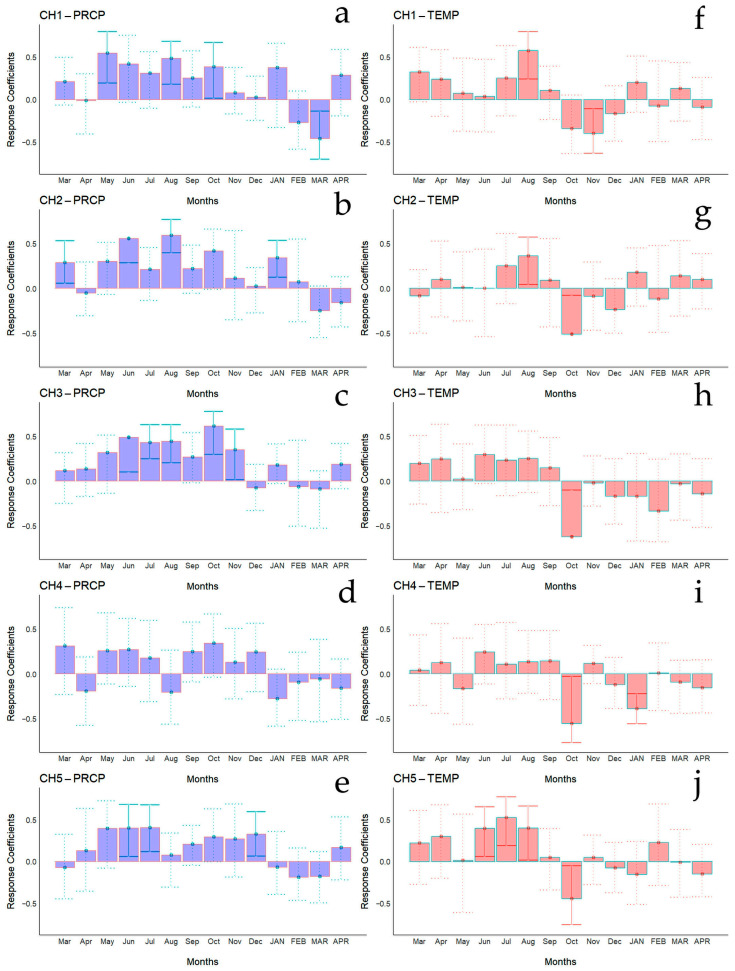
Bootstrapped correlations relating tree-ring width indices to monthly climate variables (PRCP, total rainfall—(**a**–**e**); Temp, mean temperature—(**f**–**j**)). Correlations were calculated from the previous March to the current April, and significant (*p* < 0.05) values are shown with continuous error lines. Chilean plantations: CH1 (Peñuelas), CH2 (Cahuil), CH3 (Paredones), CH4 (Manquimiliu), and CH5 (Pastene). Monthly climatic variables from the previous and current years are abbreviated with lowercase and uppercase letters, respectively.

**Figure 6:**
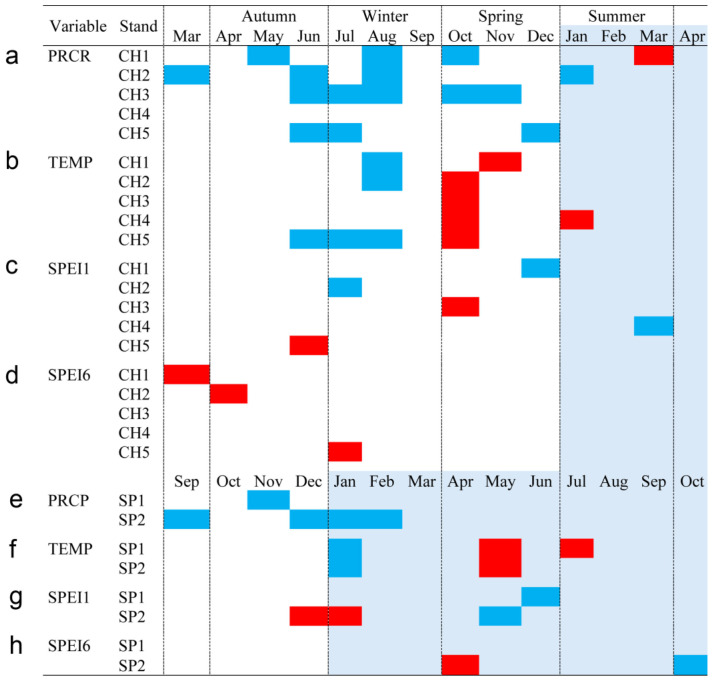
Monthly climatic variables and drought index significative correlation occurrence (*p* < 0.05) by site, month, and season. Red: significant negative correlation; Blue: significant positive correlation; light blue (current year); white (previous year). Spanish stands: SP1 (Doñana) and SP2 (Sierra Morena). Chilean plantations: CH1 (Peñuelas), CH2 (Cahuil), CH3 (Paredones), CH4 (Manquimiliu), and CH5 (Pastene). Climatic variables: total rainfall (PRCP), mean temperature (TEMP), one-month drought index (SPEI1), and six-month drought index (SPEI6) for Chile (**a**–**d**), and Spain (**e**–**h**).

Bootstrapped correlations relating tree-ring width indices to monthly climate variables for the Spanish and Chilean plantations are represented in Figure 4 and Figure 5, respectively, showing differences among populations and countries. For clarity, a summary of significant correlations between RWI and monthly climatic variables by sites is presented in Figure 6. For the Spanish plantation, a significant positive correlation was observed between RWI and November of the previous year’s rainfall (Figure 6e), whereas RWI was positively correlated with January and negatively with May and July’s mean temperature of the current year (Figure 6f). For Chilean plantations, rainfall exhibited positive correlations with RWI in 14 out of 50 months of the previous year, and in 1 out of 20 months of the current year (Figure 6a). Mean temperature was positively correlated in CH1, CH2, and CH5 in 5 out of 30 months of the previous year and none in the current year. By contrast, a significant negative correlation was found in all sites, in 5 months of 50 of the previous year and in 1 month of 12 of the current year (Figure 6b).

**Figure 7:**
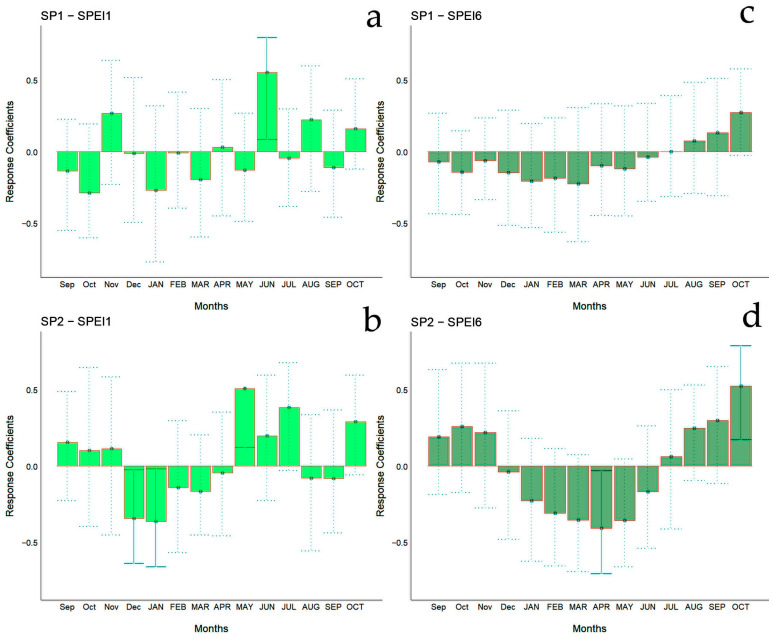
Bootstrapped response correlation analysis relating tree-ring width indices to SPEI-1 (**a**,**b**) and SPEI-6 (**c**,**d**) calculated at one- and six-month temporal resolutions. Correlations were calculated from September of the previous year to October of the current year. Significant (*p* < 0.05) values are shown with continuous error lines. Spanish stands: SP1 (Doñana) and SP2 (Sierra Morena). Monthly climatic variables from the previous and current years are abbreviated with lowercase and uppercase letters, respectively.

**Figure 8:**
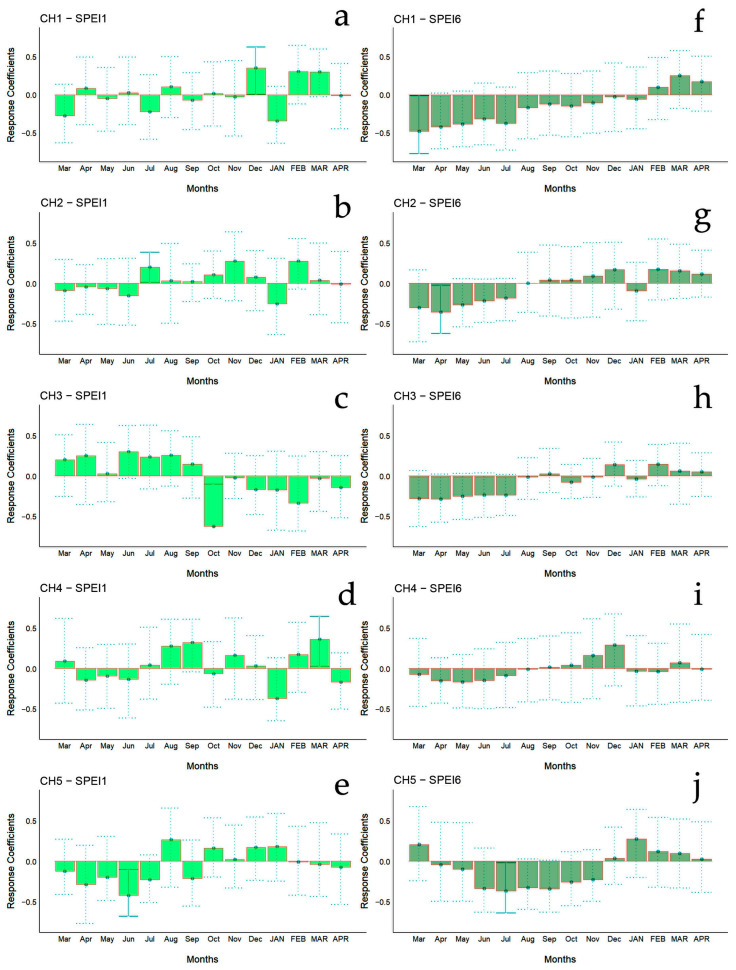
Bootstrapped response correlation analysis relating tree-ring width indices to SPEI calculated at one- (**a**–**e**) and six-month (**f**–**j**) temporal resolutions. Correlations were calculated from the previous March to the current April. Significant (*p* < 0.05) values are shown with continuous error lines. Chilean plantations: CH1 (Peñuelas), CH2 (Cahuil), CH3 (Paredones), CH4 (Manquimiliu), and CH5 (Pastene). Monthly climatic variables from the previous and current years are abbreviated with lowercase and uppercase letters, respectively.

The bootstrapped response correlation analysis relating standardized tree-ring width indices to the SPEI for the Spanish stands is shown in Figure 7 and for the Chilean plantations in Figure 8. For the Spanish stands, significant positive correlations were observed between RWI and the current year’s May and June (spring) SPEI_1_, and only for SP2 was a significant negative correlation between RWI in December of the previous year and January of the current year’s SPEI_1_ observed (Figure 6g). With respect to SPEI_6_, while SP1 showed no correlation, SP2 displayed a significant negative correlation in April of the current year and a positive correlation in October of the current year (Figure 6h).

For Chilean plantations, in terms of SPEI_1_, significant positive correlations were found in CH1 and CH4 in December of the previous year and in March of the current year (beginning and end of summer), respectively, while for CH2 it was during July of the previous year (winter) (Figure 6c); significant negative correlations were found in the CH3 and CH5 sites in October and June of the previous year (mid-spring and end of autumn, respectively) (Figure 6c). The SPEI_6_ showed negative correlations in the CH1, CH2, and CH5 sites, particularly from March to July of the previous year (autumn to winter) (Figure 6d).

### 3.3. Comparing Growth between Countries and between Forest Types

**Figure 9:**
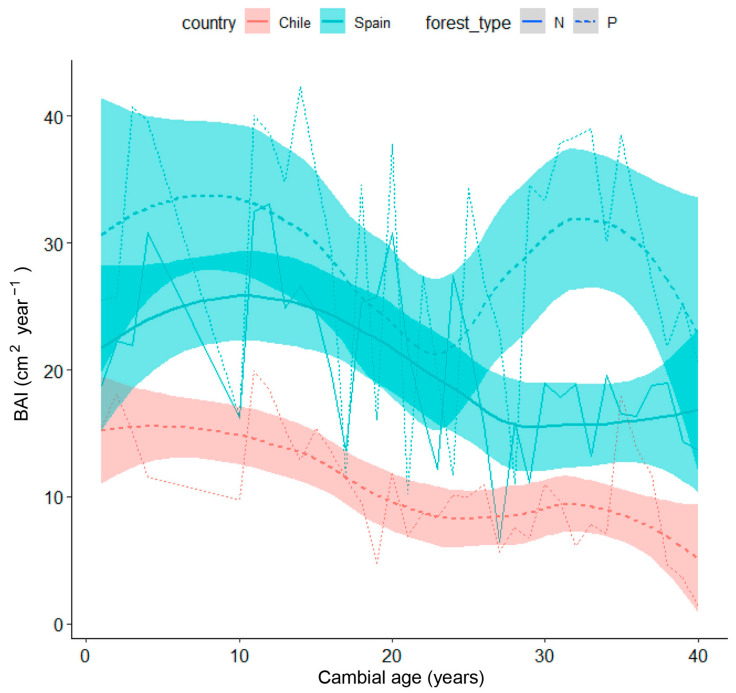
Loess Regression of the mean curves of site-grouped BAI according to tree age in stone pine in Spain (light blue lines and shaded areas) vs. Chile (pink lines and shaded areas). The shaded areas represent the higher and lower limits for the mean value of each forest type corresponding to each country. Abbreviations of forest type: N (solid line), natural; P, (dashed line) planted.

Mean curves of site-grouped BAI according to tree age (cambial year) in natural and planted stands from Spain and Chile are presented in Figure 9, showing how BAI differently decreased in both countries as trees aged and enlarged. In Chilean plantations, BAI showed a progressively decreasing trend, while in Spanish stands, it showed higher values, particularly in the planted site, but also higher year-to-year variability, probably associated with drought events.

## 4. Discussion

### 4.1. Growth Patterns

Characterizing the growth of *P. pinea* in native and exotic Mediterranean habitats is interesting in light of the challenges posed by the increasing climatic variability. In particular, the analysis of tree growth responses to climate variability in natural and planted stands in Chile and Spain provides relevant information to implement appropriate management strategies under challenging scenarios. Diameter increments in both countries showed inter-annual variability, as already reported for the species in other countries, including Syria [37], Turkey [38], Italy [8,39], and the entire Mediterranean region [9].

In general, we found lower BAI values in Chilean plantations than in the Spanish natural and planted stands. Because the limiting factor for growth is water, this difference could be attributed to an inferior drought adaptation in Chile than in the native forests and plantations from southern Spain, since the genetic material used to establish those plantations comes from reduced genetic origins [40]. Therefore, for new afforestation in non-native environments, the use of seeds from provenances with a more isohydric (water-saver) strategy towards drought stress is recommended [41].

In our study, the BAI of Chilean plantations was more similar to that of the natural Spanish stand than to the BAI of the Spain plantation. However, the Spanish plantation presented more variability than the Spanish natural stand, suggesting that plantations would be more sensitive to climate change than natural stands [42]. Natural or planted stone pine stands will be threatened by the more arid conditions induced by climate change, as reported by several authors [43], most probably increasing their susceptibility to pests and pathogens [44].

The decreasing growth trend in Chilean plantations detected here seems to be affected by the lack of management, with high densities having been kept over time; however, the low variability on plantation density in Spain and Chile calls for future studies addressing this management variable. Thinning could contribute to the reduction in vulnerability, especially in heavy treatments [45].

### 4.2. Growth Response to Climate

In Southern Europe, severe aridity is expected in the coming decades [46], intensifying drought stress in semi-arid environments [47]. This aridification trend will pose additional stress to stone pine stands because of severe hydric limitations and increasing temperatures in the studied Spanish stands. This climatic trend was also reported for central–southern Chile [48], outside the species’ natural distribution area. Therefore, alterations in the function and productivity of stone pine plantations are also foreseeable. Furthermore, dense plantations may be more vulnerable to drought stress than native forests [49,50], suggesting a potential susceptibility of Chilean plantations that should be monitored in the coming decades.

The decline in growth trends in *P. pinea* plantations has become a significant concern, particularly in the Mediterranean region where this species is native and widely cultivated. Several studies have documented this decline, attributing it to a combination of climatic changes, pest outbreaks, and anthropogenic pressures. A previous study showed that increased temperatures and reduced rainfall during the critical growing season have led to significant reductions in the species’ radial growth; the study highlighted a correlation between severe drought events and decreased tree growth, with notable reductions observed during the droughts of the early 2000s [51]. Pests and diseases have also contributed to the decline in *P. pinea* growth; for instance, it has been demonstrated that infestations by pine processionary moth (*Thaumetopea pityocampa*) have become more prevalent due to mild winters that enhance the insect’s survival and reproduction rates [52]. In addition, unsustainable forestry practices such as poor thinning have degraded the quality of *P. pinea* plantations [53]. Those factors underscore the extent of growth decline. In fact, in a long-term analysis, it has been found that *P. pinea*’s annual growth rates have decreased by approximately 20% over the past three decades in Italy [54]. Similarly, data from the Spanish National Forest Inventory reveal a consistent decline in basal area increment across several *P. pinea* plantations, with some regions reporting reductions of up to 30% in recent years [55].

Climate–growth relationships were characterized in Italian stone pine populations. The authors found regional variability and suggested that detecting differences in growth responses to site-specific climate patterns may aid in the selection of appropriate climate change mitigation strategies [8]. We detected differences between countries in the timing of significant correlations between tree-ring width indices and rainfall. Peaks occurred between autumn and spring in the Spanish natural stand, and between autumn and summer in the Spanish plantation, whereas in the Chilean plantations, it occurred during the whole year. In the case of mean temperature, in both countries, correlations were positive in winter and negative in spring and summer.

Stands located in both countries showed different responses to rainfall, temperature, and SPEI. Growth was more sensitive to climate, particularly to drought, in Chilean plantations than in the Spanish stands. Significant positive correlations between RWI and SPEI_1_ were found in both countries, whereas in Chilean plantations, the SPEI_6_ presented negative correlations. The correlation between SPEI and RWI showed different patterns between and within countries.

In stone pine, a previous study conducted in coastal areas of Italy and Greece reported that SPEI performed better than rainfall as a short-term driver for tree growth under dry climate, suggesting that temperature plays a secondary role as compared to rainfall [43]. In our study, the opposite was found for the Chilean plantations, with rainfall performing better than either SPEI_1_ or SPEI_6_. The same authors indicated that rainfall accumulated over 1 to 2 years in dry sites and over 3 to 6 years in wet sites is a key driver of stone pine radial growth, which is probably linked to fluctuating water table levels [43]; thus, future studies including rainfall accumulated over 1 to 6 years could provide new insights. 

A stone pine isohydric (drought-avoiding) strategy and xylem plasticity were found to provide a competitive advantage under moderate water shortage compared to other pines [56]. No differences have been reported in the growth responses to drought between natural and planted *P. pinea* forests, but resistance to drought was reported to be higher in natural forests than in plantations [42].

A negative impact of summer temperature was found only in one out of five Chilean plantations (CH4) and in the Spanish plantation (SP1), showing a pattern similar to a previously reported one [42]. It should be noted that canopy dieback and drought-induced mortality are currently affecting some stands in the SP1 site, characterized by poor access to shallow water pools (JJC, pers. comm.). The different responses of RWI to both rainfall and temperature in Chilean plantations suggest site-specific climate patterns, with a trend in greater control by climate in arid sites than in humid sites, as previously reported [8]. Site-specific variables, such as slope, soil type, tree density, and tree genetic origin, may either buffer [57] or exacerbate climate effects on tree growth. 

A study carried out in different European countries showed that productivity in the driest site was low and explained by consecutive years of rainfall signals [43], suggesting that tree growth was sustained mostly by soil moisture stored in the top layers from recent rainfall, a typical behavior of shallow-rooted conifers [58]. This behavior may explain the species’ vulnerability to drought stress, particularly under low rainfall, with the consequent drop in water table levels. Overall, our studied plantations did not show higher sensitivity to climate variability than the natural stand and, therefore, may be useful to assist in mitigating climate change.

Both naturally regenerated and planted stone pine stands are under threat due to the increasing occurrence and severity of droughts, a trend that will continue in the medium and long term [41]. To cope with those challenges, adequate management is crucial to preserve stone pine stands, including stand density reduction through thinning to reduce competition [57], fertilization, or even irrigation in extreme cases [59]. Regarding patterns in climate–growth relationships, the relevance of using multi-annual climatic signals in explaining growth variability reported by recent studies [43] suggests the convenience of conducting other analyses besides BAI, such as resilience indices.

## 5. Conclusions

We found heterogeneity in growth responses to climate within and between countries and forest types. In particular, *P. pinea* plantations did not show higher sensitivity to climate than the natural stands and, therefore, may be useful to assist in mitigating climate change. However, both natural and planted stands will be threatened by the more arid conditions caused by climate warming.

The comparative analysis of the growth of *P. pinea* plantations revealed differences influenced by environmental conditions and forest management in the two regions. In both countries, *P. pinea* trees are well adapted to the Mediterranean climate characterized by hot, dry summers and mild, wet winters but are facing growth challenges due to increasing drought stress. To enhance the sustainability and productivity of *P. pinea* plantations in Chile, our results suggest implementing adaptive management strategies that account for regional climatic conditions, including the optimization of management practices. Establishing comprehensive monitoring systems to continually assess tree growth, health, and productivity in plantations could be essential for making informed management decisions and adapting practices to a global change context.

## Figures and Tables

**Table 1:** Site characteristics of the *Pinus pinea* stands sampled in Chile and Spain. Abbreviations: forest type: N, natural; P, planted.

Country	Site	Location		Forest				Soil and Climate			
		Latitude	Longitude	Density (Trees ha^−1^)	Timespan	Age at Sampling (Years)	Type	Textural Class	Annual Rainfall (mm)	Average Temp. (°C)	Description
Spain	Doñana	37°2′ N	6°35′ W	180	1964–2017	54	P	Sandy	572	17.4	Mediterranean oceanic
	Sierra Morena	37°59′ N	4°48′ W	250	1927–2021	95	N	Sandy	518	17.9	Xeric Mediterranean
Chile	Peñuelas	33°11′ S	71°29′ W	833	1977–2017	40	P	Loam	384	13.6	Warm-summer Mediterranean with winter rain and coastal influence
	Cahuil	34°30′ S	72°00′ W	400	1987–2016	33	P	Loam	497	14.1	Warm-summer Mediterranean with winter rain and coastal influence
	Paredones	34°42′ S	71°53′ W	625	1985–2017	35	P	Sandy clayloam	551	14.5	Warm-summer Mediterranean with winter rain
	Manquimiliu	36°19′ S	72°31′ W	625	1993–2017	24	P	Loam	816	13.2	Warm-summer Mediterranean with winter rain
	Pastene	38°12′ S	72°60′ W	625	1984–2017	33	P	Loam	1072	10.9	Warm-summer Mediterranean with winter rain

Doñana (SP1); Sierra Morena (SP2); Peñuelas (CH1); Cahuil (CH2); Paredones (CH3); Manquimiliu (CH4); Pastene (CH5).

**Table 2:** Dendrochronological statistics of the *Pinus pinea* stands sampled in Chile and Spain. Abbreviations: BAI, mean basal area increment for the whole period; BAI_20_, mean basal area increment in the last 20 years; CG, cumulative radial growth; AC, first-order autocorrelation coefficient; MS, mean sensitivity; Rbar, mean correlation between trees; EPS, Expressed Population Signal. Mean ± SD; tree number corresponds to the number of sampled trees, and core number is presented in parentheses.

Country	Site (Code)	No. Trees (No. Cores)	BAI (cm^2^ y^−1^) †	BAI_20_ (cm^2^ y^−1^) †	CG (cm) †	AC	MS	Rbar	EPS
Spain	Doñana (SP1)	16 (31)	23.9 ± 0.02	31.9 ± 0.02	22.9 ± 1.66	0.40	0.45	0.63	0.87
	Sierra Morena (SP2)	20 (39)	13.6 ± 0.01	25.3 ± 0.01	20.8 ± 1.16	0.67	0.39	0.49	0.85
Chile	Peñuelas (CH1)	20 (39)	9.4 ± 0.01	9.9 ± 0.01	14.5 ± 0.91	0.81	0.42	0.56	0.86
	Cahuil (CH2)	20 (39)	10.9 ± 0.01	12.3 ± 0.01	12.5 ± 0.94	0.88	0.41	0.53	0.86
	Paredones (CH3)	20 (40)	12.3 ± 0.01	14.4 ± 0.01	11.3 ± 0.93	0.71	0.39	0.48	0.85
	Manquimiliu (CH4)	18 (36)	14.3 ± 0.01	16.9 ± 0.01	12.2 ± 1.22	0.69	0.35	0.71	0.89
	Pastene (CH5)	15 (30)	12.5 ± 0.01	15.2 ± 0.02	14.2 ± 1.13	0.66	0.34	0.76	0.90

†: mean ± standard error

## Data Availability

The raw data supporting the conclusions of this article will be made available by the authors upon request.

## Supplementary Materials

The following supporting information can be downloaded at: https://www.mdpi.com/article/10.3390/biology13080628/s1, Figure S1. Fitted diameter growth curves by forest types and study sites, and derived diameter growth rates of *Pinus pinea* at Spain and Chile. Forest type: N, Natural; P, Planted. Table S1. ANOVA statistics for basal area increment (BAI), basal area increment in the last 20 years (BAI_20_) and cumulative radial growth (CG).
