# Supplementary material for: Dendrochronological Analysis of Pinus pinea in Central Chile and South Spain for Sustainable Forest Management
Source: Biology (Basel). 2024 Aug 17;13(8):628. doi: 10.3390/biology13080628 (PMC11352134; doi:10.3390/biology13080628)
Supplement: Supplementary file 1 [file biology-13-00628-s001.zip › biology-3123804-supplementary.pdf]

Suplementary material

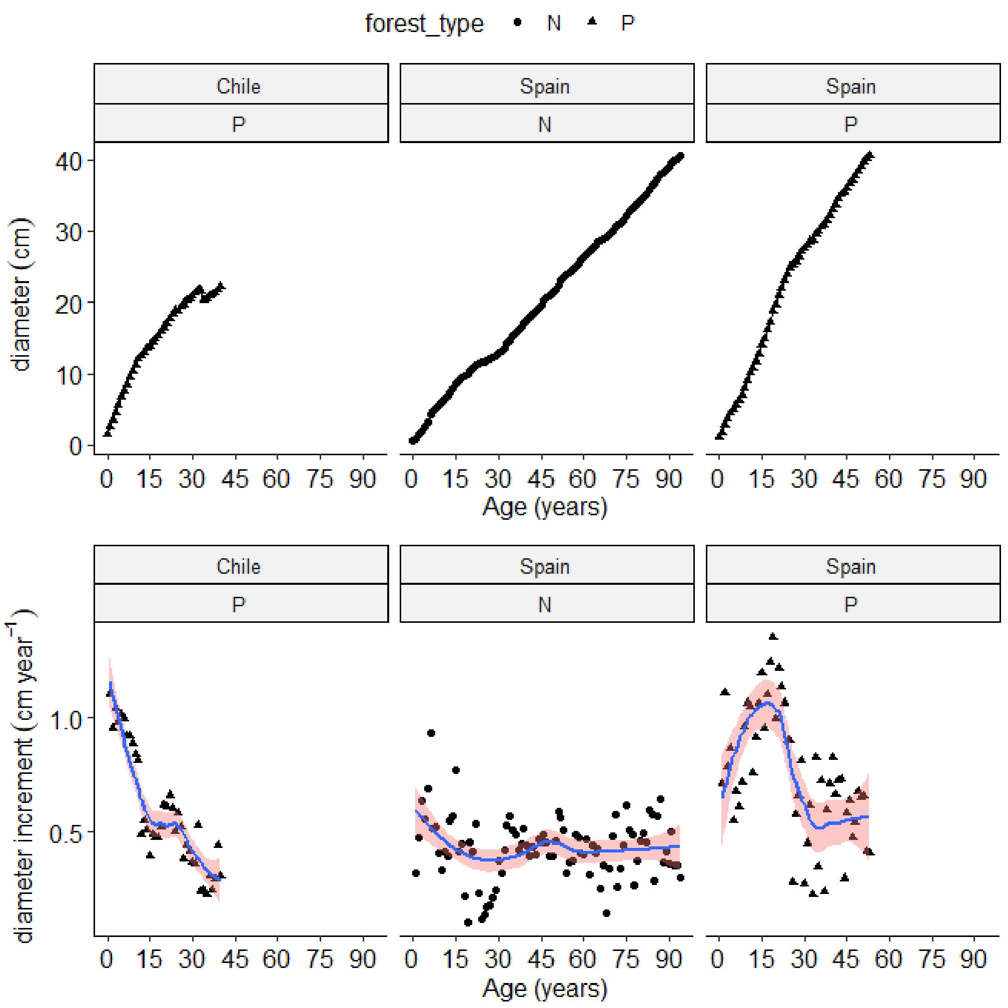

Figure S1. Fitted diameter growth curves by forest types and study sites, and derived diameter growth rates of *Pinus pinea* at Spain and Chile. Forest type: N, Natural; P, Planted.

Table S1. ANOVA statistics for basal area increment (BAI), basal area increment in the last 20 years (BAI_{20}) and cumulative radial growth (CG).

	BAI		*BAI_{20}*		*CG*	
	F	*p*	F	*p*	F	*p*
Country	35.94	<0.001	174.70	<0.001	59.434	<0.001
Forest type	53.38	<0.001	12.00	<0.001	1.76	0.186
Site	1.592	0.176	4.12	<0.001	0.39	0.813
